# 206. Using Genome-Wide Association Analysis of Pneumococcal Isolates to Identify Novel Gene Variants in Central Nervous System Infection

**DOI:** 10.1093/ofid/ofac492.283

**Published:** 2022-12-15

**Authors:** John Michael Sanchez, Nicole L Pershing, Shannon Nielsen, Aurelie Kapusta, Hillary Crandall, Anne J Blaschke

**Affiliations:** University of Utah School of Medicine, Cedars-Sinai Medical Center, Salt Lake City, Utah; University of Utah School of Medicine, Salt Lake City, Utah; University of Utah School of Medicine, Salt Lake City, Utah; University of Utah School of Medicine, Salt Lake City, Utah; University of Utah School of Medicine, Salt Lake City, Utah; University of Utah School of Medicine, Salt Lake City, Utah

## Abstract

**Background:**

*Streptococcus pneumoniae* is the leading cause of meningitis in children and is associated with significant morbidity and mortality. Despite conjugate pneumococcal vaccines that have otherwise been successful in reducing invasive disease caused by vaccine serotypes, the incidence of pneumococcal meningitis remains relatively unchanged. Using genome-wide association, we identified sequence elements associated with pneumococcal central nervous system (CNS) infection.

**Methods:**

From 1996 to 2018, 366 clinical pneumococcal isolates were collected from pediatric patients and paired with clinical metadata. All isolates underwent whole-genome sequencing and *in silico* serotyping. Fifty-four clinical isolates were from children with CNS infection. Sequence element enrichment analysis was performed using elastic net and linear mixed modeling statistical methods to find genetic variants associated with CNS infection. We identified associated genes by mapping statistically associated variants to a serotype 19F reference *S. pneumoniae* genome.

**Results:**

CNS isolates spanned 22 unique serotypes; the most common were 22F (n = 6), 7F (n = 5), 18C (n = 4), and 35B (n = 4). We identified two intergenic sequence variants and one intragenic sequence variant significantly associated with CNS infection (p < 1.48 × 10^-7^). The top intragenic candidate in our analysis was located within cpsC, a gene involved in regulation of capsule synthesis. The CNS infection-associated cpsC variant was found in serotypes 22F (n = 1), 18C (n = 4), and 14 (n = 2).

**Conclusion:**

The diversity of pneumococcal isolates associated with CNS infection suggests the importance of genetic determinants beyond capsular serotype. We identified a number of novel microbial genetic variants significantly associated with pneumococcal CNS infection across serotypes, including a coding mutation in cpsC. Functional characterization of cpsC variants is needed to inform mechanisms contributing to pneumococcal CNS virulence.

**Disclosures:**

**Anne J. Blaschke, MD, PhD**, BioFire Diagnostics/Biomerieux: Advisor/Consultant|BioFire Diagnostics/Biomerieux: Grant/Research Support|BioFire Diagnostics/Biomerieux: IP licensed to BioFire Diagnostics through the University of Utah and royalties received through the University of Utah related to the FilmArray|Merck: Advisor/Consultant.

